# A new and efficient procedure to load bioactive molecules within the human heavy-chain ferritin nanocage

**DOI:** 10.3389/fmolb.2023.1008985

**Published:** 2023-01-13

**Authors:** Rosanna Lucignano, Ilaria Stanzione, Giarita Ferraro, Rocco Di Girolamo, Carolina Cané, Angela Di Somma, Angela Duilio, Antonello Merlino, Delia Picone

**Affiliations:** Department of Chemical Sciences, University of Naples Federico II, Naples, Italy

**Keywords:** ferritin nanocages, reversible ferritin disassembly, drug encapsulation, disassembly/reassembly nanocage protocols, human ferritin nanocarriers

## Abstract

For their easy and high-yield recombinant production, their high stability in a wide range of physico-chemical conditions and their characteristic hollow structure, ferritins (Fts) are considered useful scaffolds to encapsulate bioactive molecules. Notably, for the absence of immunogenicity and the selective interaction with tumor cells, the nanocages constituted by the heavy chain of the human variant of ferritin (hHFt) are optimal candidates for the delivery of anti-cancer drugs. hHFt nanocages can be disassembled and reassembled *in vitro* to allow the loading of cargo molecules, however the currently available protocols present some relevant drawbacks. Indeed, protein disassembly is achieved by exposure to extreme pH (either acidic or alkaline), followed by incubation at neutral pH to allow reassembly, but the final protein recovery and homogeneity are not satisfactory. Moreover, the exposure to extreme pH may affect the structure of the molecule to be loaded. In this paper, we report an alternative, efficient and reproducible procedure to reversibly disassemble hHFt under mild pH conditions. We demonstrate that a small amount of sodium dodecyl sulfate (SDS) is sufficient to disassemble the nanocage, which quantitatively reassembles upon SDS removal. Electron microscopy and X-ray crystallography show that the reassembled protein is identical to the untreated one. The newly developed procedure was used to encapsulate two small molecules. When compared to the existing disassembly/reassembly procedures, our approach can be applied in a wide range of pH values and temperatures, is compatible with a larger number of cargos and allows a higher protein recovery.

## 1 Introduction

Different biological systems form supramolecular complexes with suitable size and attractive functions through spontaneous self-assembly of monomeric proteins. This process is driven by chemical and physical forces, such as van der Waals, electrostatic and hydrophobic forces, magnetic interactions, and entropic effects (Men et al., 2015). Nature’s idea of self-assembly can also be exploited in several bionanotechnological applications. In fact, biological supramolecular structures like virus capsids, cellular membranes, protein complexes and artificial cells, are being developed for application in engineering, medicine, and material sciences. Along these lines, viral capsids, and some protein complexes, such as ferritins and heat shock proteins, are particularly attractive models: they are readily produced in large quantities by heterologous expression *in vitro*, have well-characterised atomic structures, are usually monodisperse in solution, and are amenable to chemical and biological modifications (He and Marles-Wright, 2015).

Ferritins (Fts) are ubiquitous proteins in nature and are the subject of many research studies focused on their applications in bionanotechnology. The main role of Fts is to protect cells from the damage related to the Fenton reaction (Chakraborti and Chakrabarti, 2019). In fact, under oxidizing conditions, free Fe(II) ions produce reactive oxygen species that can damage the cells. Fts are also able to store a high amount of iron within a hollow core acting as an iron deposit for the cells. In eukaryotes and in some bacteria Ft is made-up of 24 subunits that naturally self-assemble to form a nanocage structure. A deep analysis of the interactions driving the self-assembly of ferritin cages has been reported (Huard et al., 2013). The cage has octahedral symmetry, with external and internal diameters of 12 nm and 8 nm, respectively (Theil, 1987). The human protein (hFt) contains two types of subunits: L chains (light, MW ∼19 kDa), and H chains (heavy, MW ∼21 kDa), whose relative amount depends on the specific tissue (Harrison and Arosio, 1996). H chains exert ferroxidase activity and have a high affinity for the TfR1 transferrin receptor, over-expressed on the surface of different cancer cell lines (Li et al., 2010). L chains are responsible for iron storage.

Homopolymeric Fts constituted by human H chains (hHFt) are considered very promising nanosystems for drug delivery (Li et al., 2010). The advantages of using these proteins as drug carriers are related to a series of features (Zhen et al., 2013). hHFt is biocompatible: it is already present in the human body; thus, it is recognized by the immune system, and its administration can avoid undesired reactions and side effects. It is soluble in the bloodstream, and it can easily deliver hydrophobic molecules to the final target without the use of toxic organic co-solvents. Like other Fts, it displays high thermal and chemical stability and the capability to undergo controlled disassembly and reassembly *in vitro*. Moreover, the surface of Ft can be chemically or genetically functionalized to obtain proteins for specific targeting. Despite its potential, drug encapsulation in human Ft nanocages still presents some drawbacks. Indeed, the main protocols used to load drugs within Fts are based on a pH switch: hHFt is disassembled at extreme pH values (pH = 2.0 or 12.0/13.0), and then it is reassembled in the presence of the selected drug, restoring a physiological pH. The main disadvantage of this protocol is that the process is not fully reversible, leading to the formation of amorphous aggregates. In fact, only 10%–25% of disassociated protein molecules could reassemble into a properly folded protein nanocage (Zhang et al., 2021). Moreover, strong acidic or alkaline pH values can damage both the nanocage protein and the cargo. Alternative approaches involving the use of chemical denaturants such as high urea concentration, guanidinium hydrochloride or a combination of high temperature and denaturants (Zhang et al., 2020), did not lead to an increase in encapsulation capacity (Lei et al., 2016). Furthermore, the high concentration of denaturant needed to dissociate the nanocage or the high temperatures can disrupt the cargo molecules. Alternative strategies have been developed to overcome these limitations. Indeed, it has been demonstrated that drug loading can be improved under pressure and that some Fts can disassemble and reassemble at neutral pH in the absence/presence of cations (Macone et al., 2019).

Here, we describe an easy and alternative protocol to encapsulate drugs within hHFt, based on the use of a low concentration of sodium dodecyl sulfate (SDS) to disassemble the protein nanocage. The protocol allows an efficient recovery of hHFt and high-performance encapsulation of molecules of different chemical compositions and sizes, ranging from metal complexes to polypeptides.

## 2 Materials and methods

### 2.1 Recombinant production of hHFt

The H chain of human Ft (hHFt) was expressed in *E. coli* using a synthetic plasmid (Pet-12a, Novagen) and purified from the soluble fraction according to the protocol described in (Santambrogio et al., 2000), with minor modifications. Briefly, the pellet corresponding to 1 L of culture collected upon induction with 0.4 mM IPTG was suspended in 120 mL of lysis buffer 20 mM Tris-HCl pH 7.4 and sonicated after the addition of anti-protease agents (anti protease Cocktail, Sigma Aldrich, one tablet per 30 mL) and 75 μg/mL of DNase and RNase solutions (Sigma Aldrich). The supernatant was heated at 75°C for 10 min, centrifuged to remove the precipitated proteins, and loaded on a DEAE column pre-equilibrated with 20 mM Tris-HCl pH 7.4. The column was eluted with a linear gradient of NaCl (0–1.0 M) in the same buffer at a flow rate of 1.0 mL/min. Protein presence in the collected fractions was monitored by UV absorbance at 280 nm and SDS-PAGE. The fractions containing the pure protein were pooled and loaded on a Sepharose 6B column (2.5 × 24 cm) equilibrated and eluted in 20 mM Tris-HCl pH 7.4. The fractions containing the assembled hHFt nanocage were concentrated by ultrafiltration (cut-off 100.000 Da), and stored at 4°C.

Protein purity and folding were assessed by SDS-PAGE and circular dichroism (CD) spectroscopy.

### 2.2 Disassembly/reassembly protocols

The purified hHFt was disassembled by SDS treatment. The optimization of the protocol is reported in the Results section. To allow the reassembly, the SDS concentration was decreased below 0.01% using two different approaches, *i.e.,* by alternating steps of ultrafiltration and dilution with pure buffer or by extensive dialysis, as described in detail in the Results section. For comparison, disassembly/reassembly protocols following literature procedures based on pH switch were also applied (Yang et al., 2007; Pontillo et al., 2016), starting from four identical aliquots of hHFt (0.5 mg/mL, 1 mL). Briefly, the pH of two aliquots was slowly adjusted to 1.5 or 2.0 by gentle addition of 0.1 M HCl (hHFt_pH2->7.4_) and the pH of the two remaining was slowly adjusted to 12.0 or 13.0 by gentle addition of 0.1 M NaOH (hHFt_pH13->7.4_). The four solutions were incubated for 2 h at room temperature, then the pH was restored to 7.4 by 30 times dilution with 20 mM Tris-HCl pH 7.4. After incubation overnight at 4°C to allow the reassembly, the samples were concentrated by ultrafiltration on Centricon tubes (cut-off 100.000 Da).

Assembly and disassembly states of hHFt samples were evaluated by non-denaturing (native) PAGE on a 6% polyacrylamide gel for the separation, using 25 mM Tris/glycine pH 8.4 as running buffer (Petrák and Vyoral, 2001). The gels were run for 3.5 h at a constant voltage of 100 V, at 4°C. Coomassie Brilliant Blue G-250 was used as staining agent.

### 2.3 Drug encapsulation

The disassembly/reassembly protocol was applied to encapsulate two small molecules, the [Ru(bipyridyl)_3_]^2+^ metal complex (henceforth named Ru1), kindly provided by Prof. P. Manini (Dpt. of Chemical Sciences, University of Naples Federico II) and the 13-residue antimicrobial peptide dubbed TRIL (FVKWFKKFLTRIL), an analogue of Temporin-L (Di Somma et al., 2020) purchased from BioFab Research.

#### 2.3.1 Ru1 encapsulation

800 µL of Ru1 solution (1 mM in 20 mM Tris-HCl pH 7.4) were added to 2 mg of hHFt (0.5 mg/mL, 4 mL, dissolved in the same buffer) dissociated in the presence of 0.1% SDS (hHFt_SDS_), according to the protocol optimized as reported in the Results section. The mixture was stirred for 30 min at room temperature, then the sample was diluted 10 times with the buffer (20 mM Tris-HCl pH 7.4) to decrease the SDS concentration, and the solution was incubated at 4°C overnight. Free Ru1 and residual SDS were removed by ultrafiltration on Centricon tubes with cut-off of 100.000 Da and extensive washing with Tris-HCl buffer up to a final volume of 550 μL. The protein concentration before and after the treatments was assessed by using the Bradford assay kit (Sigma), using BSA as a standard (Bradford, 1976). The amount of Ru1 encapsulated within hHFt was before estimated by the absorbance at 452 nm, using a molar extinction coefficient of 1.46 × 10^4^ (M^−1^ cm^−1^) (Li et al., 2016) and then measured by multi-element analysis performed by the Inductively Coupled Plasma - Mass Spectrometer (ICP-MS Aurora M90, Bruker, Germany) conducted at the laboratory of Analytical Chemistry for the Environment of the University of Naples Federico II. Nitric acid (HNO_3_, 69% v/v Ultratrace^@^ ppb-trace analysis grade) was provided by Scharlau (Barcelona, Spain). Multi-component solution of 30 elements (10 mg/L each one) was of ultrapure grade for ICP, TraceCERT^®^ and was purchased by Merck (Darmstadt, Germany); ruthenium solution (1.000 mg/L) was of ultrapure grade for ICP, TraceCERT^®^ and was purchased by ROMIL.

The analysis was performed in Normal Sensitivity mode. All standards used for analysis in ICP-MS were prepared in HNO_3_ solution (2%, v/v). The internal standards were ^89^Y and ^115^In for both calibration curve and sample analysis. All the analyses were performed as triplicates.

The aggregation state of the nanocages reassembled in the presence of Ru1 (Ru1-hHFt), was assessed by native PAGE, while the protein secondary structure was evaluated by circular dichroism spectroscopy.

#### 2.3.2 TRIL encapsulation

For the encapsulation of the peptide, a slightly different procedure was adopted, using a higher protein concentration in the dissociation step and a 1/1 cargo/ferritin subunit molar ratio. 180 µL of a TRIL solution (2.0 mg/mL, equivalent to 1.2 mM, dissolved in 20 mM Tris-HCl pH 7.4) were added to 5.6 mg of hHFt (11.2 mg/mL, 500 µL) disassembled with 0.1% SDS, as previously described, and incubated overnight at room temperature. The solution was diluted 10 times with 20 mM Tris-HCl at pH 7.4 and incubated overnight at 4°C to allow the reassembly. The protein solution was concentrated by ultrafiltration using Centricon tubes with cut-off 100.000 Da and extensively washed with the buffer to remove any residual trace of SDS and the free peptide. Also in this case, the aggregation state of the nanocages reassembled in the presence of TRIL (TRIL-hHFt) was assessed by native PAGE. The presence of the peptide in the high molecular weight fraction (*i.e.,* > 100.000) was assessed by SDS-PAGE and mass spectrometry, as described below.

### 2.4 Spectroscopic measurements

UV−vis absorption spectra were recorded at 25°C using a 1.0 cm optical path-length quartz cells on a JASCO V-750 UV−vis spectrophotometer in the range of 240–700 nm, using protein concentration of 0.25 mg/mL in 20 mM Tris-HCl buffer pH 7.4. Other experimental parameters were bandwidth 2.0 nm, scanning speed 200 nm/min, data pitch 1.0 nm.

Far UV-CD spectra were recorded on a Jasco J-715 spectropolarimeter equipped with a Peltier thermostatic cell holder using a 0.1 cm path length quartz cell. Spectra were registered at 25°C in the range of 190–250 nm at a protein concentration of 0.05 mg/mL in 20 mM Tris-HCl buffer pH 7.4. Measurements were recorded with a time constant of 2 s, a 2 nm bandwidth, and a scan rate of 50 nm/min. Three scans for each spectrum were acquired.

### 2.5 Transmission electron microscopy (TEM) characterization

Samples for TEM analysis were prepared by placing a drop of a protein solution (typical concentration 0.5 mg/mL) on a carbon-coated copper TEM grid and allowing the solvent (water) to evaporate. To enhance the contrast, negative staining with 1.5% phosphotungstic acid (PTA) solution at pH 7.0 was carried out by depositing a drop of PTA solution on the grid containing the sample for 4 min (contact between sample and PTA) and then the excess fluid was drained off with filter paper. The grid was allowed to dry, and images were collected using a FEI TECNAI G2 S-twin apparatus operating at 120 kV (LaB_6_ source).

### 2.6 Crystallization, X-ray diffraction data collection, structure resolution and refinement

hHFt restored after the SDS dissociation (hHFt_SDS,_ see results section), Ru1-hHFt and TRIL-hHFt systems were crystallized by hanging-drop vapor diffusion method at 293 K. The drops were prepared by mixing 1 μL of 5–10 mg/mL Ru1-hHFt or TRIL-hHFt solution with 1 μL of the precipitant solution (2.0 M MgCl_2_, 0.1 M bicine at pH 9.0) and equilibrated against the precipitant solution (0.5 mL) at 20°C. The crystals grow within a week.

X-ray diffraction data were collected at 100 K on a Pilatus detector at XRD2 beamline of the Elettra synchrotron, Trieste, Italy, using a X-ray wavelength of 1.0 Å. Data were processed and scaled in the F432 space group using AutoProc (Vonrhein et al., 2011) to 1.52, 1.57, and 2.30 Å resolution for the hHFt_SDS_, Ru1-hHFt and TRIL-hHFt systems obtained in SDS. To carry out the data collection at 100 K, crystals were frozen in liquid nitrogen upon exposure to a cryoprotectant solution containing the reservoir and 25% glycerol. Details on data collection statistics for these crystals are reported in Supplementary Table S1. The phase problem was solved by molecular replacement method using the hHFt structure deposited in the Protein Data Bank (PDB) under the accession code 5N27 (Ferraro et al., 2017) as starting model. Refinement was carried out using Refmac5.0 (Murshudov et al., 1997) and model visualization and building using Coot (Emsley et al., 2010).

The structures were refined to R_factor_ and R_free_ values of 0.153/0.190, 0.161/0.190, and 0.171/0.223, respectively. Refinement statistics are reported in Supplementary Table S1. The final models were validated using the PDB validation server and deposited in the PDB under the accession codes 8A5N, 8A2M and 8A2L for hHFt_SDS,_ Ru1-hHFt and TRIL-hHFt, respectively. All figures were drawn with PyMOL (DeLano Scientific LLC, San Carlos, CA, United States).

### 2.7 MALDI spectra

A MALDI Voyager-DE STR spectrometer from Applied Biosystems was used for the acquisition of MALDI spectra. The sample co-crystallizes with an excess of matrix, α-cyano-4-hydroxycinnamic acid, equal to 10 mg/mL on a metallic support and it is ionized by a laser pulse (*λ* = 337 nm) in the MALDI source. The analysis was performed in positive mode using the TOF analyser in reflection mode and a mass range of 200–5,000 m/z.

## 3 Results and discussion

### 3.1 Nanocage disassembly/reassembly

**FIGURE 1:**
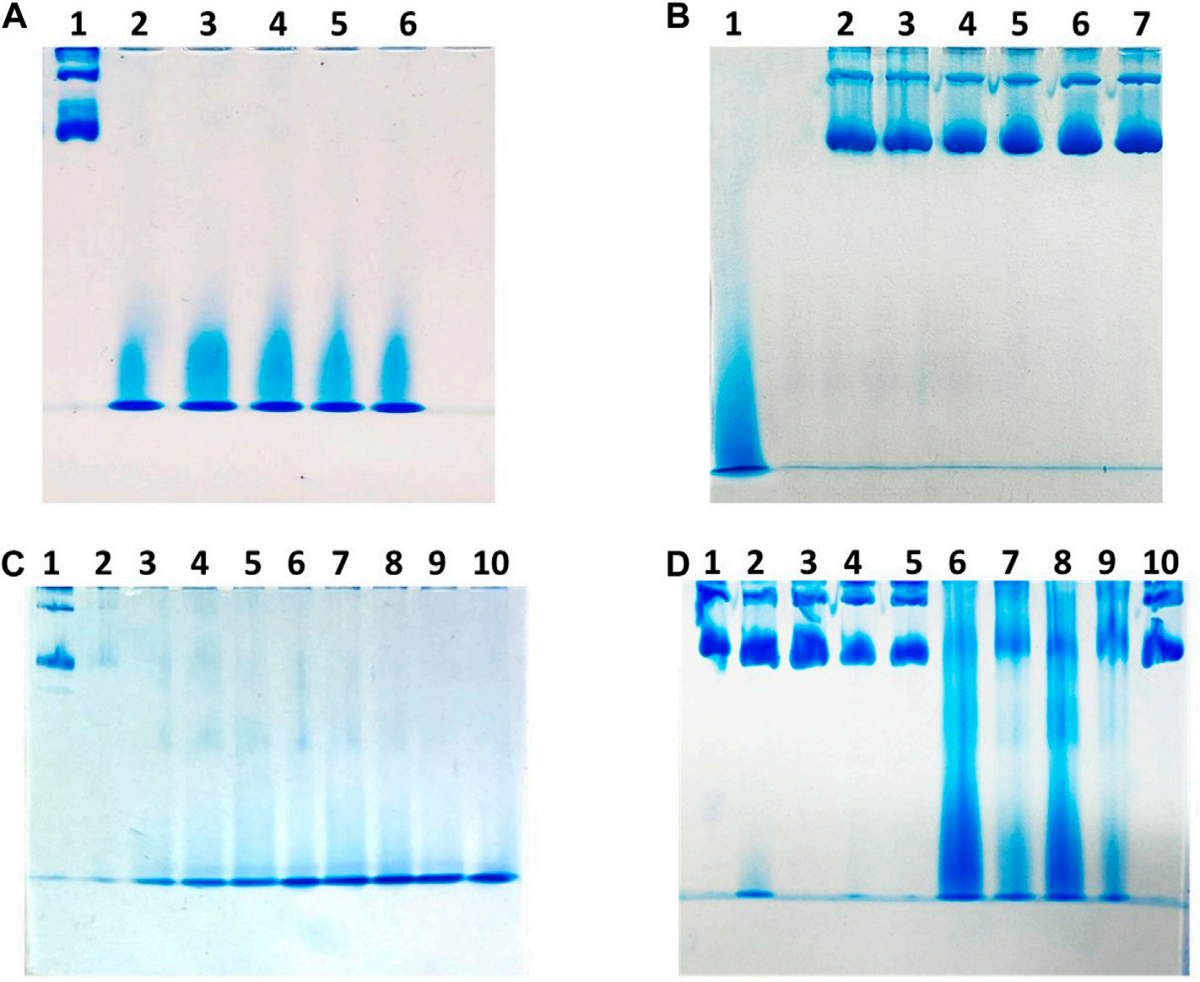
Gel electrophoresis under native conditions of hHFt to evaluate the assembly state upon different treatments. **Panel (A) *lane 1*** untreated hHFt; from ***lane 2*** to ***lane 6*** hHFt incubated with 1.0%, 1.5%, 2.0%, 2.5% and 3.0% SDS, respectively. **Panel (B) *lane 1*** untreated hHFt; from ***lane 2*** to ***lane 6*** hHFt incubated with 1.0%, 1.5%, 2.0%, 2.5% and 3.0% SDS after SDS removal by dialysis against 20 mM Tris-HCl pH 7.4. **Panel (C) *lane 1*** untreated hHFt; from ***lane 2*** to ***lane 10*** hHFt incubated with 0.01%, 0.02%, 0.03%, 0.04%, 0.05%, 0.06%, 0.07%, 0.08%, and 0.09% SDS, respectively. **Panel (D) *lane 1***: untreated hHFt; ***lane 2*** hHFt_pH12_; ***lane 3*** hHFt_pH 12->7.4_ (reassembled); ***lane 4*** hHFt_pH 13_; ***lanes 5*** hHFt_pH 13->7.4_ (reassembled); ***lanes 6*** hHFt_pH 1.5_; ***lane 7*** hHFt_pH 1.5->7.4_ (reassembled); ***lane 8*** hHFt_pH 2_; ***lane 9*** hHFt_pH 2->7.4_ (reassembled); ***lane 10*** hHFt_SDS 0.1%_ (reassembled).

With the aim to provide an alternative protocol to encapsulate drugs within recombinant hHFt, we developed an efficient procedure to disassemble hHFt nanocages using low concentrations of SDS, followed by dilution with pure buffer to allow the reassembly. The results obtained with this new protocol were compared with those obtained using standard methods consisting of the disassembly of the protein nanocage upon exposure to very acidic or alkaline pH and to its reassembly at neutral pH. The aggregation state of the protein upon different treatments was assessed by native PAGE electrophoresis (Figure 1). In a first experiment, five identical samples of 3 mg/mL hHFt dissolved in 20 mM Tris-HCl buffer pH 7.4 were incubated overnight in the presence of SDS at final concentrations of 1.0%, 1.5%, 2.0%, 2.5%, and 3.0%. Aliquots of each sample were analysed on native gel (Figure 1A, ***lanes 2–6***). The comparison with the untreated sample (***lane 1***) revealed that all the tested SDS concentrations can completely disassemble the hHFt nanocages, as only a single band with a very high electrophoretic mobility was detected. After SDS removal by 10-fold dilution and extensive dialysis against 20 mM Tris-HCl pH 7.4, the samples were concentrated by ultrafiltration (cut-off 100.000 Da) to the initial volume and loaded on the gel (Figure 1B). Only the bands corresponding to the untreated sample (***lane 1***) were detected in all the lanes, indicating that the hHFt nanocage is correctly reconstituted, *i.e.,* the disassembly process is reversible. A following experiment was carried out in similar conditions, but using lower concentrations of SDS, from 0.1% to 0.9%. Also in this case, PAGE under non-denaturing conditions revealed the efficient disassembly/reassembly of hHFt in all the explored experimental conditions (data not shown). Encouraged from this result, we evaluated the possibility of a further decrease of SDS percentages, from 0.01% to 0.09%. The PAGE analysis (Figure 1C) reveals significant differences in the dissociation, as the nanocages are only partially dissociated at the lowest SDS concentrations, *i.e.,* from 0.01% to 0.07% (Figure 1C, ***lanes 2–8***), while full dissociation occurs only in the presence of 0.08% and 0.09% SDS. Based on these data, in the following experiments we selected 0.1% SDS to allow an extensive nanocage dissociation, while to remove the SDS and to achieve an efficient recovery of the reassembled form we used a 10 times dilution followed by overnight incubation at 4°C. Residual SDS was removed by extensive washing with pure buffer on Centricon tubes with cut-off 100.000 Da. The efficiency of the disassembly/reassembly procedure is illustrated in Supplementary Figure S1. In a further experiment, we decided to compare the disassembly/reassembly procedure based on the treatment with 0.1% SDS with the disassembly/reassembly protocols based on the pH switch, following the procedures described in detail in the experimental section. The results of the native gel, reported in Figure 1D, revealed that upon treatment both at pH 12.0 and 13.0 (***lane 2*** and ***lane 4***, respectively) a large fraction of the protein migrates still like the untreated sample (***lane 1***), suggesting that the alkaline treatment allows only a partial disassembly of the nanocages. On the other hand, the acidic treatment allows a more efficient disassembly with respect to the alkaline treatment, as the band of the reference protein almost disappears (***lanes 6*** and ***8*** for the samples disassembled at pH 1.5 and 2.0, respectively). Still, the disassembly process is not complete, as also indicated by the presence of an intense smear along both the lanes. As described in the experimental section, to allow the reassembly the samples were diluted with pure buffer, restoring the pH to 7.4, and incubated overnight at 4°C. Then, before the native PAGE analysis, all the samples were concentrated to the initial volumes by ultrafiltration on Centricon tubes with cut-off 100.000 Da. In both cases, *i.e.,* upon incubation at pH 1.5 and 2.0, the nanocage band reappears after increasing the pH up to 7.4 (***lanes 7*** and ***9***), but no full recovery is achieved, since significant amounts of high-mobility species were still detected on the gel. In contrast, only the band corresponding to the 24-mer is detected in the sample reassembled after dissociation carried-out in the presence of 0.1% SDS (Figure 1D, ***lane 10***). Interestingly, while during both the treatments at alkaline pH and with SDS the protein solutions remained always clear, the protein solutions incubated at acidic pH became cloudy when restoring the pH to 7.4, suggesting that a precipitation occurred. Accordingly, an estimation of the protein amount in the samples before and after the disassembly/reassembly procedures carried out by UV absorption spectroscopy showed that no significant protein loss occurred for samples treated at basic pH and with SDS, while more than 50% of the samples disassembled at acidic pH is lost.

Thus, based on the results of this comparative study, to perform a deep structural characterization of the hHFt nanocages obtained after the disassembly/reassembly procedure based on the treatment with 0.1% SDS, we prepared a new sample. Starting from 1 mL of a solution containing 1 mg of hHFt, after extensive washing with pure buffer in Centricon tubes (cut-off 100.000 Da) to remove residual SDS, the sample was concentrated to the initial volume. The final protein yield, estimated by UV absorbance, was 0.9 mg.

**FIGURE 2:**
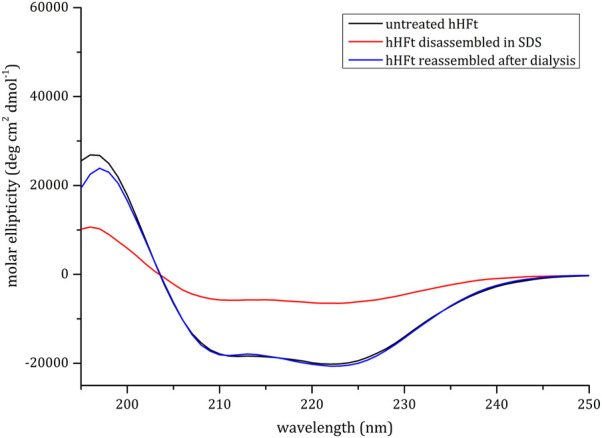
CD spectra of hHFt during the SDS treatment. ***Black line***: untreated hHFt in 20 mM Tris-HCl pH 7.4; ***Red line:*** hHFt treated with 0.1% SDS; ***Blue line***: hHFt in 20 mM Tris-HCl pH 7.4 after SDS removal.

CD spectra, reported in Figure 2, revealed a substantial persistence of α-helical structure in the hHFt sample treated with SDS even though a reduction of the secondary structure content is evident in the disassembled sample. Notably, it can be appreciated that the hHFt structure is fully recovered after SDS removal, since the curves of the untreated protein and of the restored one are overlapped.

**FIGURE 3:**
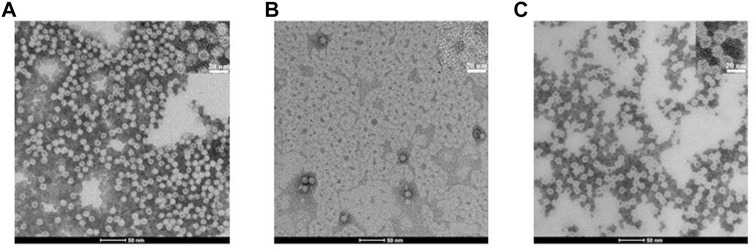
TEM bright field images ((scale bar = 50 nm, upper inset, 20 nm) of hHFt during the SDS treatment. **(A)** Reference (untreated) hHFt. **(B)** hHFt _SDS 0.1%_; **(C)** hHFt _SDS 0.1%_ reassembled after SDS removal. Samples were stained with 1.5% phosphotungstic acid. Notably, despite the use of negative staining, some hHFt nanocages appear slightly dark because of PTA absorption.

To further demonstrate that the protein cage was really disassembled and reversibly reassembled using the SDS protocol, TEM experiments were performed. The image of the untreated protein, clearly showing the presence of numerous and empty hHFt nanocages, is displayed in Figure 3A. The outer and inner diameters of the particles were 12.5 ± 0.6 nm and 7.7 ± 0.4 nm, respectively. Figure 3B shows the efficient dissociation of the nanocages produced by 0.1% SDS, since only a few residual isolated hHFt nanocages can be observed. Finally, Figure 3C demonstrates the correct reconstitution of the nanocages after SDS removal. Indeed, the hHFt reassociates with a morphology identical to that of the untreated sample with an outer diameter of 12.0 ± 0.4 and an inner diameter of 7.8 ± 0.4 nm, thus also this analysis further proved that the disassembly is completely reversible.

**FIGURE 4:**
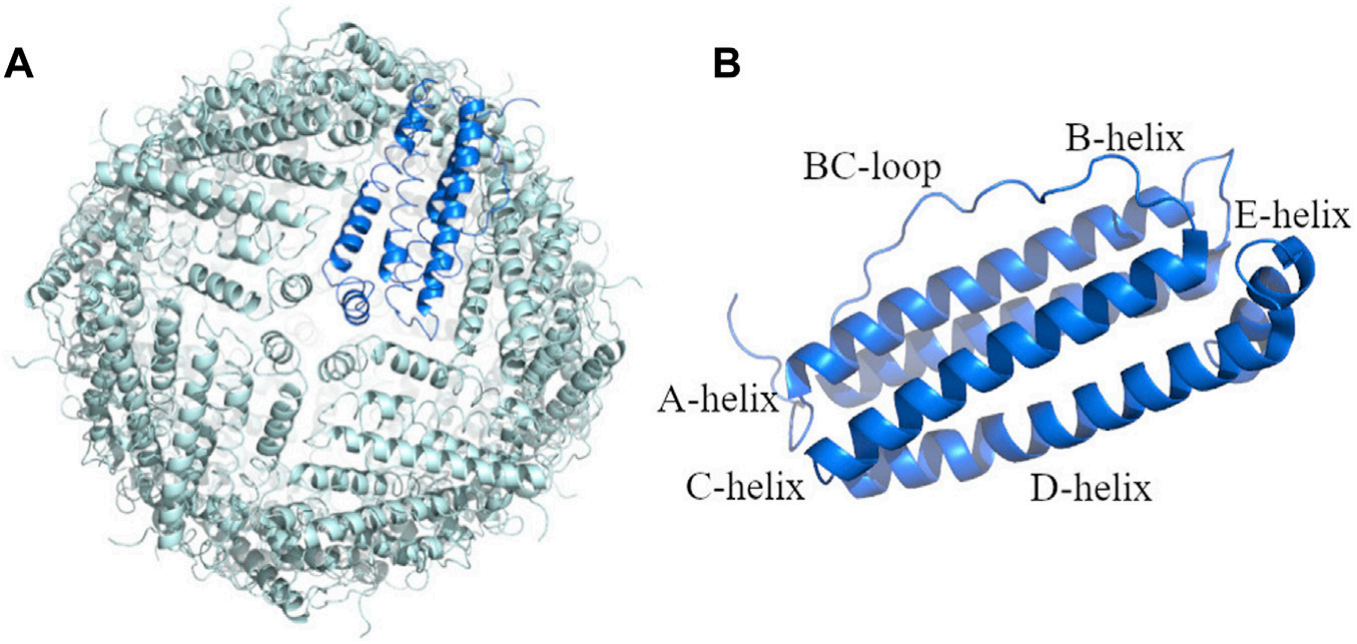
X-ray structure of the hHFt reassembled after disassembly in SDS: **(A)** the 24-mer (hHFt_SDS_); **(B)** the single chain of hHFt. The asymmetric unit of the crystal contains one single chain; the 24-mer is generated by crystal symmetry. In panel B, the four helices (A-D), the short helix at the C-terminal tail (helix E) and the BC loop are highlighted. The structure has been deposited with the PDB code 8A5N.

To verify that the hHFt reconstituted after SDS removal has the same features of the untreated hHFt, crystals of the protein after the SDS treatment (hHFt_SDS_) were grown and the X-ray structure was solved. The structure, refined to 1.52 Å resolution (Figure 4), shows that the protein correctly reassembles after the SDS treatment, retaining all the features of the untreated sample. In particular, hHFt monomers adopt the four-helix bundle structure typical of the Ft fold (Figure 4A) and assemble to form the hollow cage constituted by 24 molecules in 432 symmetry (Figure 4B).

The Cα r.m.s. deviation of the hHFt_SDS_ from that of hHFt in the starting model (PDB code 5N27) is as low as 0.61 Å. The shape, overall structure, polarity/hydrophobicity, volume, and electrostatic potential of the surface are conserved (Supplementary Table S2), thus unequivocally demonstrating that the cages correctly reassemble upon the SDS treatment.

Encouraged from the obtained results, we evaluated the possibility to use the SDS-based procedure to encapsulate two small molecules of different chemical composition, *i.e.,* the [Ru(bpy)_3_]^2+^ complex (Ru1) and a 13-residue bioactive peptide (TRIL), an analogue of the antimicrobial peptide Temporin L (Di Somma et al., 2020) in hHFt.

### 3.2 Ru1 encapsulation

**FIGURE 5:**
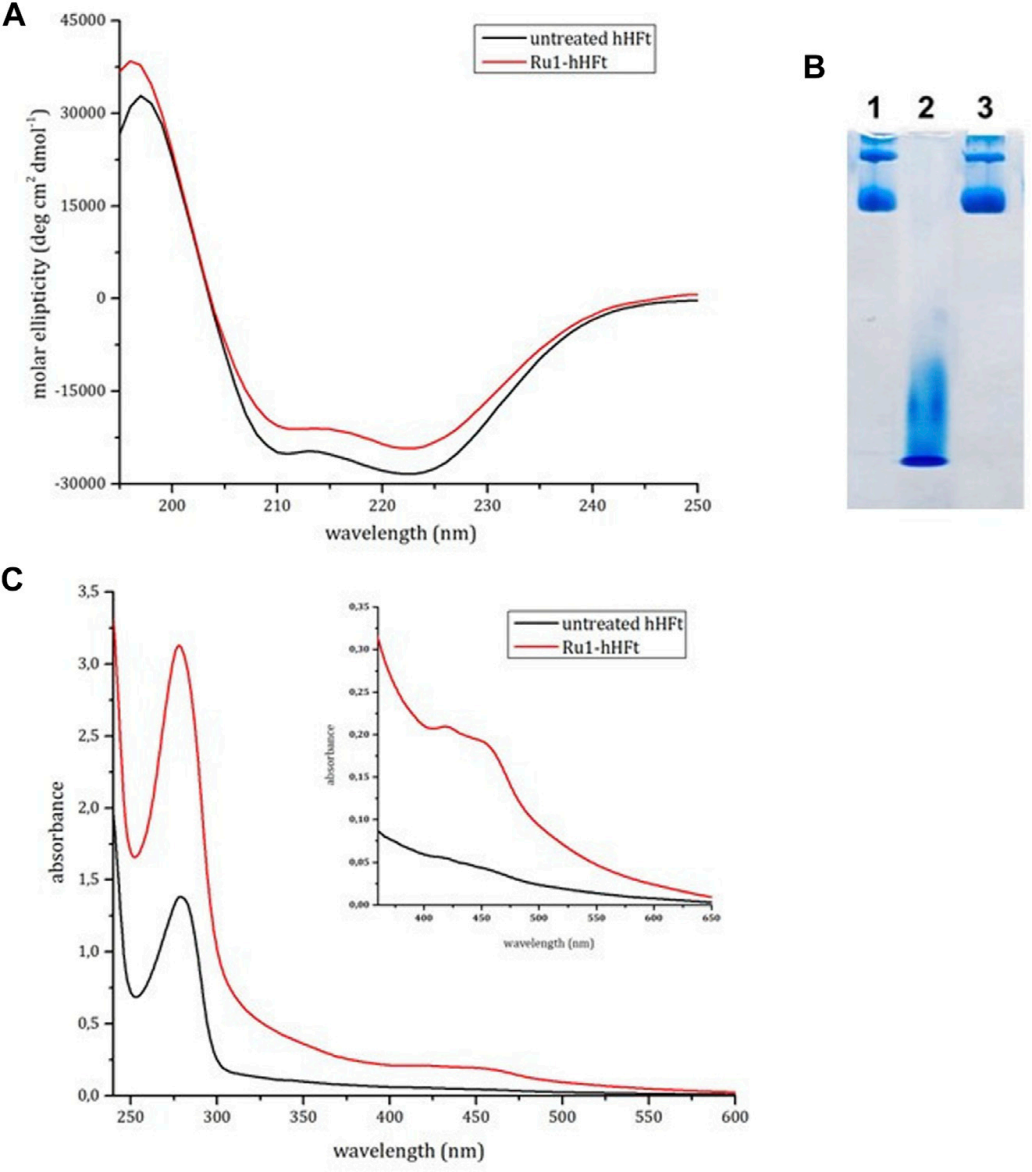
Electrophoretic and spectroscopic analysis of Ru1-hHFt. **(A)** CD spectra of reference hHFt (black line), and of Ru1-hHFt (red line). **(B)** electrophoresis under native conditions of hHFt, ***lane 1*** reference hHFt, ***lane 2*** disassembled hHFt incubated with 0.1% SDS, and ***lane 3*** Ru1-hHFt after encapsulation. **(C)** UV-vis spectra of reference hHFt (black line), and of Ru1-hHFt (red line). All the spectra were acquired in 20 mM Tris-HCl pH 7.4.

The SDS-based disassembly/reassembly protocol was used to encapsulate the metal compound Ru1 within the human H-ferritin using a 200:1 metal complex to hHFt cage molar ratio. Details of the encapsulation protocol are described in the experimental section. As indicated in Figure 5A, after the SDS treatment the protein recovers its native structure upon reassembly. Indeed, the CD profile of the Ru1-hHFt sample was almost superimposable to that of the untreated one. The successful reassembly of the nanocages was verified by native gel electrophoresis, as reported in Figure 5B. Indeed, the Ru1-hHFt sample (***lane 3***) shows the same profile as the untreated protein (***lane 1***), while ***lane 2*** indicates the efficiency of the disassembly procedure.

To estimate the amount of metal encapsulated within Ru1-hHFt, the UV-vis absorption spectrum was acquired in comparison with that of the untreated sample, at the same protein concentration. The Ru1-hHFt (Figure 5C) showed an increase of the absorbance in the 400–500 nm range, confirming the presence of the Ru compound associated with the protein sample. The Ru1 concentration estimated using the absorbance at 452 nm was 26 μM, while the protein concentration calculated using the Bradford assay was 6 μM, corresponding to a Ru1/hHFt cage molar ratio of 4:1. The effective amount of the metal complex within hHFt was assessed by ICP-MS, yielding a value of 4.03 ± 0.19. This amount is comparable with the data reported by Li et al. and Conti et al. (Li et al., 2016; Conti et al., 2022), through pH switch-based disassembly/reassembly protocols. Interestingly, the BCA assay allowed also to assess that more than 90% of protein was recovered after the complex encapsulation procedure.

Since CD spectra cannot provide indication on the correct cage reassembly of the Ru1-hHFt system (Belletti et al., 2017), the X-ray structure of this system was also solved. The structure was refined to 1.57 Å resolution to R_factor_/R_free_ values of 0.161/0.190 and compared to that of the untreated protein (PDB code 5N27) (Ferraro et al., 2017) and with hHFt_SDS._ The structural analysis (Supplementary Tables S1, S2) indicates that the overall conformation of hHFt in the Ru1-hHFt system formed in SDS is very similar to that of hHFt_SDS_ and of the untreated protein. Notably, analysis of the electron-density map reveals that the Ru complex is not directly coordinated to the protein, neither on the inner shell nor in the outer shell, as observed when another Ru complex has been encapsulated within the horse spleen ferritin nanocage (Petruk et al., 2019).

To confirm the presence of the Ru complex within the Ru1-loaded hHFt system, crystals of Ru1-loaded hHFt and of hHFt_SDS_ were dissolved and analysed spectrophotometrically. The UV-vis spectrum of Ru1-hHFt from dissolved crystals shows a small band between 300 and 350 nm, observed also in the spectrum of the protein treated with the Ru compound in solution, but not found in the spectrum of hHFt (Supplementary Figure S2). This result unambiguously demonstrates that crystals of Ru1-hHFt obtained upon the SDS-based encapsulation protocol and used for the structural determination contained the metal compound. Notably, the spectrum of dissolved Ru1-hHFt crystals does not show signals between 400 and 450 nm. The origin of this difference in the spectra is unclear, but it suggests a modification of the Ru compound structure inside the hHFt crystal, that alters its absorption properties.

### 3.3 Peptide encapsulation

**FIGURE 6:**
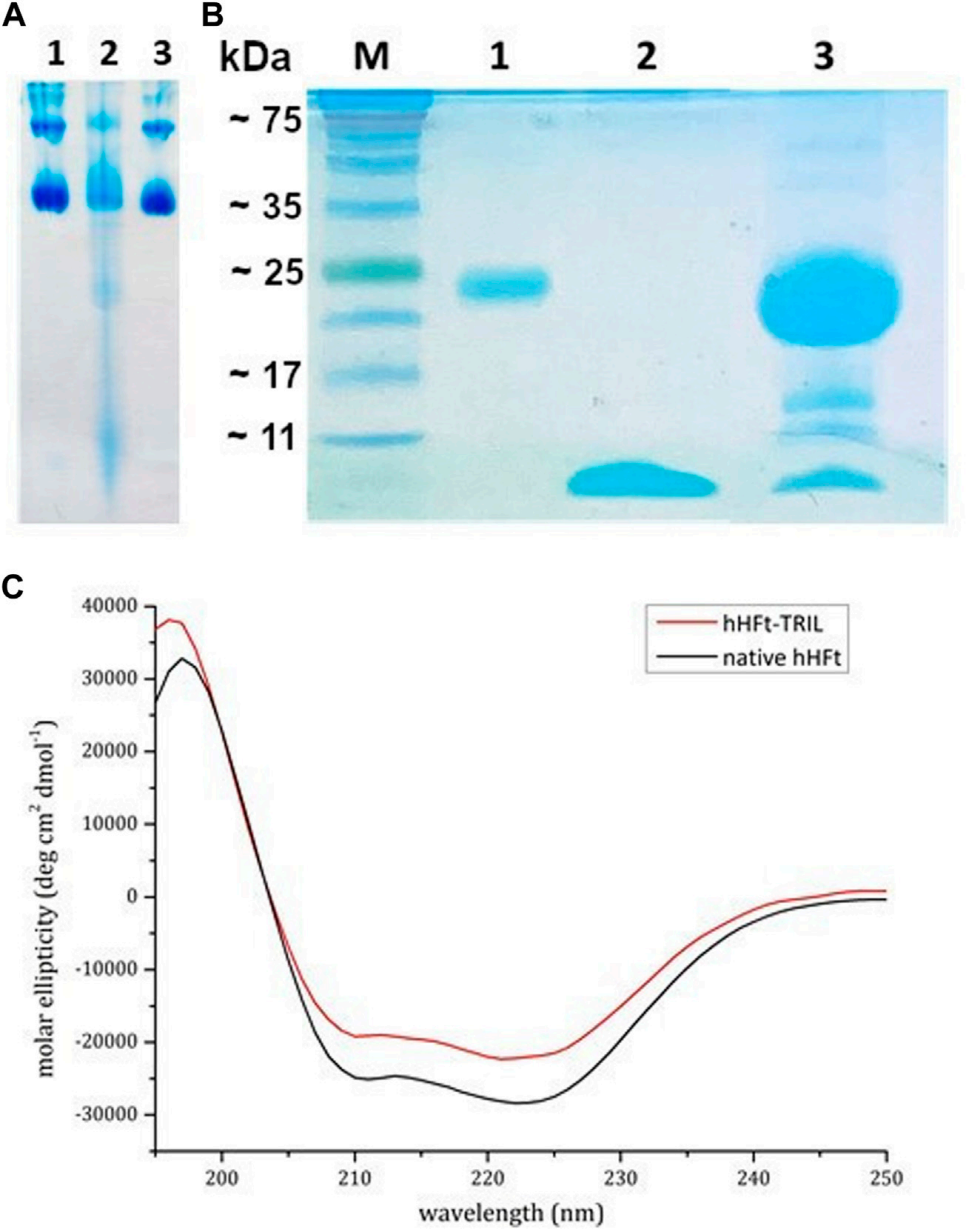
Electrophoretic and spectroscopic analysis of TRIL-hHFt: **(A)** Gel electrophoresis under native conditions of hHFt, ***lane 1*** reference hHFt and ***lane 2*** TRIL-hHFt after the encapsulation. **(B)** 15% SDS-PAGE gel of and treated samples: ***M*** proteins marker, ***lane 1*** reference hHFt, ***lane 2*** TRIL, ***lane 3*** TRIL-hHFt after the encapsulation. **(C)** CD spectra of native hHFt (black line) and of TRIL-hHFt (red line) in 20 mM Tris-HCl pH 7.4.

With the aim to verify if the developed disassembly/reassembly protocol can be used also to encapsulate peptides within the hHFt nanocage, TRIL and hHFt previously disassembled in SDS were mixed at a molar ratio of 1/1 peptide/protein subunit, as described in detail in the experimental section. Remarkably, in this procedure we used a higher protein concentration, while keeping the SDS concentration of 0.1%. Upon 10 times dilution and incubation overnight, the reassembled nanocages were concentrated by ultrafiltration using Centricon tubes with cut-off 100.000 Da and analysed by gel electrophoresis under native and denaturing conditions. The native gel (Figure 6A, ***lane 2***) reveals that, after the SDS treatment, the hHFt nanocages are disassembled but not completely converted into the highest electrophoretic mobility specie(s) observed when a much lower protein concentration is used, as multiple forms are detected. Also in this case, upon SDS dilution the nanocages are completely restored (***lane 3***), since the electrophoretic profile of the protein recovered is identical to that of the untreated one (***lane 1***).

The denaturing gel-electrophoresis (Figure 6B) of TRIL-hHFt (***lane 3***) was carried out by using both the untreated hHFt (***lane 1***) and TRIL (***lane 2***) as controls, confirming the presence of both hHFt subunit and peptide at the right molecular weights. The lowest band, corresponding to the antimicrobial peptide, was excised from the gel, and digested with trypsin. The resulting peptide mixture was directly analysed by MALDI mass spectrometry. The mass spectrum (Supplementary Figure S3) confirms the identity of the antimicrobial peptide with a 100% sequence coverage. Supplementary Table S3 reports the assignments of the mass signals recorded in the spectra.

Also, as done in the case of the Ru complex, CD spectra (Figure 6C) of TRIL-hHFt were collected. The spectra show that TRIL-hHFt possesses the same secondary structure of the untreated hHFt, thus indicating a correct protein refolding.

Finally, to obtain a structural description of the TRIL-hHFt system, crystals were grown, and X-ray diffraction data were collected. The SDS-PAGE of the dissolved crystals (Supplementary Figure S4, ***panel A***) confirmed the presence of the peptide together with that of the ferritin subunit. Also in this case, the band related to TRIL peptide was excised and analysed by MALDI mass spectrometry (Supplementary Figure S4, ***panel B*,**; Supplementary Table S3) reaching a total sequence coverage. The crystals diffract X-rays to 2.3 Å resolution, *i.e.,* a resolution significantly lower than that of the Ru1-hHFt and hHFt_SDS_ crystals that diffracted to 1.57 and 1.52 Å, respectively. The structure of TRIL-hHFt was solved and refined up to R_factor_ and R_free_ values of 0.171 and 0.223 (Supplementary Table S1). Also in this case, the protein cage is perfectly reconstituted upon the encapsulation protocol and the peptide is not visible in the electron density map. Details on the main features of the cage for the TRIL-hHFt system are reported in Supplementary Table S2. These findings indicate once again that the protein can correctly reassemble upon the SDS disassembly/reassembly protocol and suggest that the peptide does not simply interact with the outer protein nanocage surface.

## 4 Conclusion

The great potential of human ferritin nanocages as drug carrier is limited by the lack of efficient and reproducible loading procedures. The size of the potential drugs often requires reversible disassembly of the nanocage, but the high chemical and thermal stability of the protein, while representing a positive feature for the resistance of the nanocarrier, turns into a drawback for the efficient encapsulation of bioactive substances into the protein cavity. Current protocols are essentially based on a treatment at very acidic or very alkaline pH to dissociate the multimer, followed by incubation under mild conditions, *i.e.,* physiological pH (Yang et al., 2007; Pontillo et al., 2016) to allow its reassembly. However, the range of molecules that can be exposed to such extreme pH conditions without structural alterations is quite limited. Even worse, the protein shell is not completely reconstituted, as heterogeneous multimeric structures are obtained (Palombarini et al., 2020).

In this paper we have developed a new protocol that allows the quantitative disassembly and the high-yield recovery of hHFt nanocages whose structural properties are identical to those of the untreated form. At room temperature, in our explorative study based on native-PAGE analysis, we were able to define a narrow range of SDS concentrations where heterogeneous hHFt species coexist, *i.e.,* between 0.01% and 0.07% of SDS (Figure 1C). On the other hand, outside this interval we observed only a single band corresponding to small-size (high mobility) specie(s) on the highest SDS percentage side, and fully assembled, homogeneous 24mer nanocages below 0.01% SDS. Thus, given the qualitative significance of this analysis, we set-up our procedure based on a disassembly step in the presence of 0.1% SDS, followed by a simple dilution of the solution with the pure buffer, to allow the spontaneous reconstitution of the multimeric, native structure. To remove the residual SDS, we used alternating steps of ultrafiltration and dilution with pure buffer, using Centricon tubes with cut-off 100.000 Da. This procedure allows an easy and quantitative control of the protein recovery. However, single protein subunits or aggregated forms with a molecular size below 100.000 Da could be lost during the ultrafiltration procedure. Nevertheless, whenever a quantitative evaluation of the protein amount was performed, we observed 90% of recovery. Actually, the reduction of the SDS concentration below 0.1% can be achieved also by means of an extensive dialysis against the pure buffer. This procedure can be useful when big volumes of buffer are required to allow the reassembly. As a matter of fact, we applied the dialysis only to reassemble the hHFt disassembled in the presence of the highest SDS concentrations (Figures 1A, B for the disassembly and reassembly data, respectively). Qualitative evaluation of the data suggested high-yield reconstitution of the original nanocages upon SDS removal, however these experimental conditions were no longer used in our study.

The dissociation step we applied is not critical for the protein structure. Indeed, despite a substantial loss of the secondary structure indicated by CD spectroscopy, the reconstituted hHFt fully recovers its original secondary, tertiary, and quaternary structures, as indicated by different structural analyses. In particular, in the native gel we do not see any traces of intermediate size/mobility species. Interestingly, during the disassembly/reassembly steps no protein precipitation occurred and an almost quantitative recovery of the protein was achieved. Furthermore, the CD spectra of the protein recovered upon reassembly was completely overlapped with that of the reference, untreated one. No differences were observed in the electron microscopy images and also the high-resolution X-ray structure was almost identical to that of the original complex.

Our protocol was also applied to load two small molecules within the protein nanocage, since drug encapsulation in hHFt can have significant advantages, *i.e.,* it increases the solubility of the small molecules in aqueous media and/or their thermal stability, decreases the immunogenicity, allows a selective targeting towards tumor cells, and prevents other possible side effects of the drug. Using Ru1 as model, we demonstrated that the SDS-based protocol allows the encapsulation of metal complexes within hHFt nanocage. Again, the structure of the reassembled protein in the presence of Ru1 was identical to that of the untreated sample, and the BCA analysis indicated that the protein recovery was quantitative. Further studies are currently in progress to increase the cargo loading efficiency.

We also performed the encapsulation of TRIL, a 13-residue antimicrobial peptide, demonstrating that polypeptides can also be loaded within the nanocage obtaining nanoparticles amenable for further structural and functional studies. In this case, we used a higher ferritin concentration during the dissociation step, while keeping the SDS concentration identical to that of the reference experiments, *i.e.* 0.1%. Also in these conditions an extensive disassembly of the hHFt nanocages occurred, but the native gel electrophoresis reported in Figure 6A revealed the presence of some species with an electrophoretic mobility significantly lower with respect to those observed at 0.5 mg/mL nanocages concentration. Even in these conditions a successful encapsulation of the peptide was achieved, demonstrating the versatility of the proposed method, since even a not complete disassembly of the ferritin can allow the loading of small molecules. Furthermore, given the extreme sensibility of protein and peptide functionality to their correct folding, preliminary characterization of the specific molecule features, such as the influence of the SDS on their stability, and possible optimization of other experimental conditions (*i.e.,* ionic strength, pH, temperature) would be necessary.

Notably, SDS was not detected in the X-ray structures of the empty hHFt reconstituted after disassembly, nor in the nanocages loaded with Ru1 or TRIL. However, despite the extensive wash of the reassembled nanocages with pure buffer, we cannot exclude the presence of traces of SDS (below 0.01%) trapped in the bulk inside the nanocage. This SDS quantity, if any, is not expected to influence the peptide functionality, once released from the nanocage into the physiological *milieu*, *i.e.* the target cell. Further studies are in progress to demonstrate the absence of toxic effects of the reconstituted nanocages.

Overall, the obtained data open interesting perspectives to develop new nanoparticles which combine the properties of hHFt with those of the cargo molecules, with synergistic effects.

We believe that the advantages of the proposed procedure, *i.e.,* the easy protocol, the versatility of some experimental conditions such as the protein/SDS ratio, the potential application at different pHs and ionic strength, the homogeneous reconstitution of the hHFt nanocages after dissociation and the highly efficient protein recovery will allow the effective encapsulation of different classes of molecules and boots the development of new nanoparticles with important innovative applications.

## Data Availability

The datasets presented in this study can be found in online repositories. The names of the repository/repositories and accession number(s) can be found below: http://www.wwpdb.org/, 8A5N http://www.wwpdb.org/, 8A2M http://www.wwpdb.org/, 8A2L.
