# Supplementary material for: A new and efficient procedure to load bioactive molecules within the human heavy-chain ferritin nanocage
Source: Front Mol Biosci. 2023 Jan 13;10:1008985. doi: 10.3389/fmolb.2023.1008985 (PMC9880187; doi:10.3389/fmolb.2023.1008985)
Supplement: Supplementary file 1 [file DataSheet1.pdf]

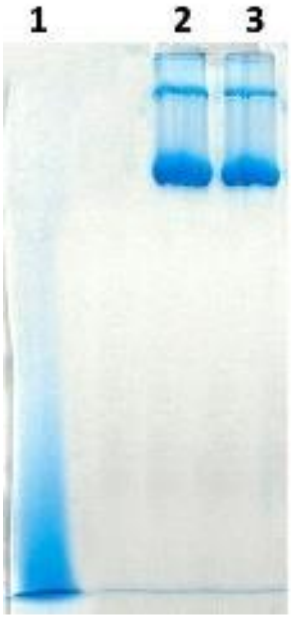

Figure S1: Gel electrophoresis under native conditions of hHFt to evaluate the disassembly/reassembly process after incubation with SDS: ***(lane 1)*** hHFt incubated with 0.1% SDS; ***(lane 2)*** hHFt reassembled after SDS; ***(lane 3)*** untreated hHFt.

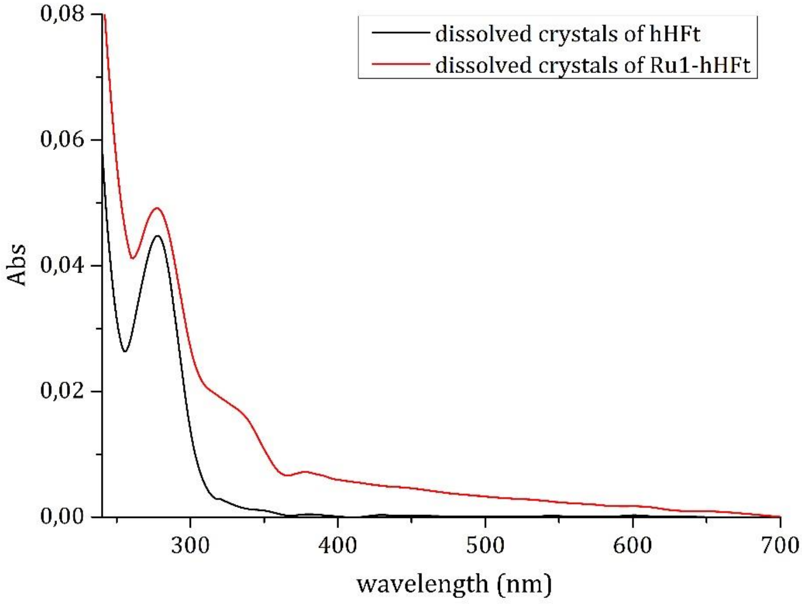

Figure S2: UV-Vis spectra of dissolved crystals (in water) of hHFt disassembled and reassembled upon SDS treatment (hHFt, black line) and of Ru1-hHFt (red line).

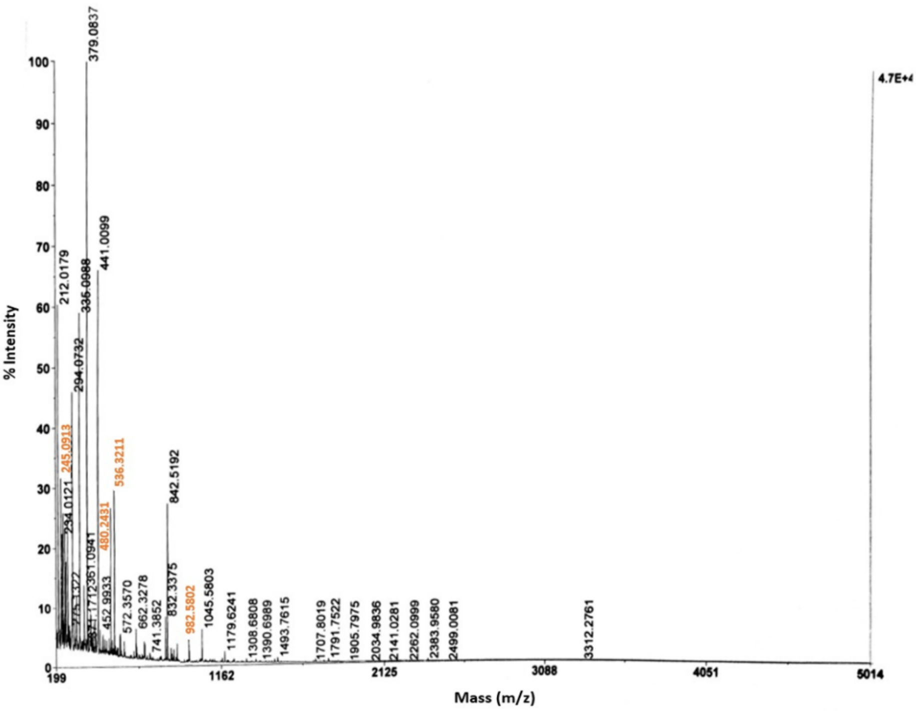

Figure S3*:* Assessment of the encapsulated peptide identity by mass spectrometry. The spectrum shows the relative abundance of ions as a function of the mass/charge (m/z) ratio. In orange the signals corresponding to peptides. The low intensity of the signals reflects the low amount of peptide obtained after extraction from the gel and hydrolysis. Notably, when a very low concentration of sample is available, like in this case, it is very common to observe some other peaks arising from the matrix (379.08, 441.00 m/z), the staining used (842.51 m/z) and the keratin (1045, 1179, 1308, 1707, 1791, 3312 m/z), often present as contaminants.

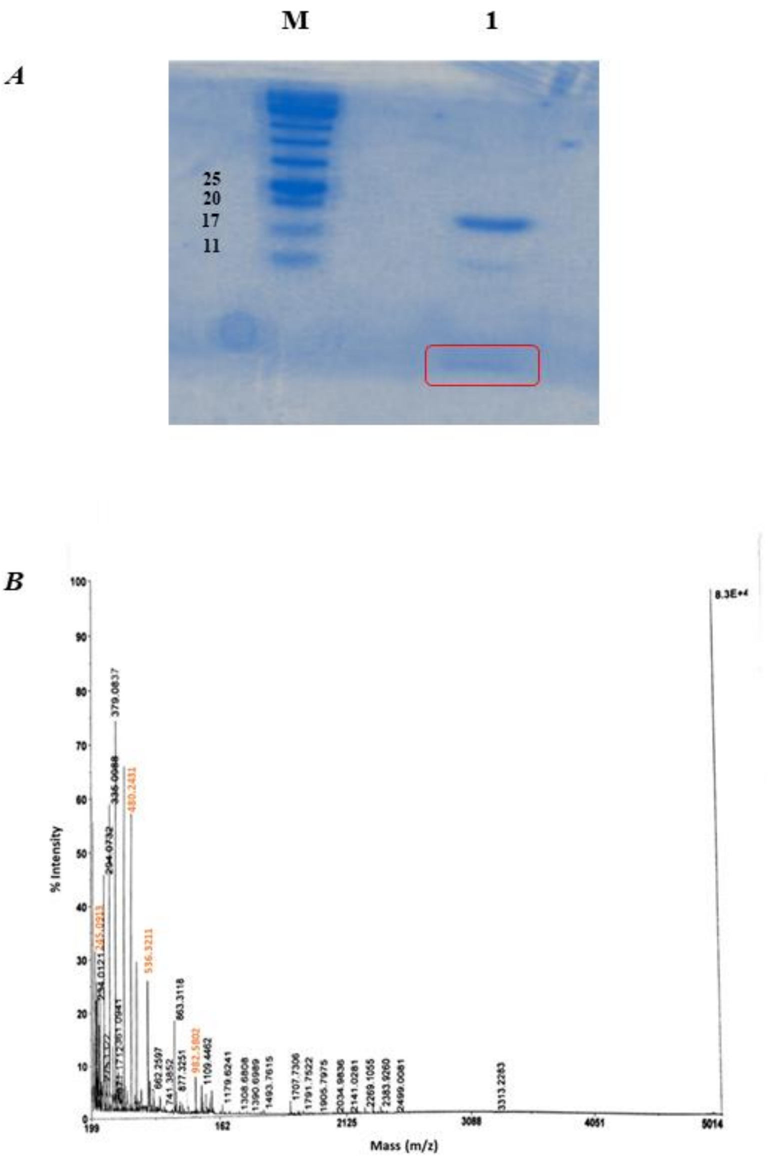

Figure S4. Assessment of the presence of TRIL in the dissolved crystal of TRIL-hHFt. *Panel A*: 15 % SDS-PAGE of the dissolved crystal (***lane 1***) showing the presence of the peptide (in red box) with the expected molecular weight; *Panel B*: MALDI mapping analysis of the red band excised from the gel. The spectrum shows the relative abundance of ions as a function of the mass/charge (m/z) ratio. In orange the signals corresponding to the peptide, whose attribution is reported in Table S3.

Table S1. Data collection and refinement statistics for $hHFt_{SDS}$, Ru1-hHFt and TRIL-hHFt formed using the SDS disassembly/reassembly protocol.

	**$hHFt_{SDS}$**	**Ru1-hHFt**	**TRIL-hHFt**
Protein			
Data collection			
Space group	F432	F432	F432
a=b=c (Å)	183.92	183.77	183.06
α=β =γ (°)	90.0	90.0	90.0
Resolution range (Å)	106.18- 1.52 (1.55-1.52)*	106.10- 1.57 (1.59-1.57)*	105.69-2.30 (2.34-2.30)
Total number of observations	5748728 (294577)	2574356 (120863)	1695326 (83457)
Total number unique reflections	41264 (2030)	37411 (1836)	12141 (607)
Completeness (%)	99.5 (100.0)	99.4 (100.0)	99.9 (100.0)
Redundancy	139.3 (145.1)	68.8 (65.8)	139.6 (137.5)
†Rmerge (%)	0.185 (4.027)	0.248 (3.076)	0.709 (4.363)
Rmeas	0.185 (4.040)	0.250 (3.099)	0.711 (4.379)
Rpim	0.016 (0.334)	0.030 (0.380)	0.059 (0.371)
Average I/σ(I)	41.2 (2.4)	15.0 (2.1)	13.0 (2.2)
$CC_{1/2}$	1.00 (0.859)	0.998 (0.846)	0.998 (0.827)
Refinement			
Resolution range (Å)	106.18-1.52	105.69-1.57	105.69-2.30
N. of reflections (working set)	2056	2571	829
N. of reflections (test set)	153	137	603
R-factor/R-free (%)	0.153/0.190	0.161/0.190	0.171/0.223
N. of atoms	1920	1848	1635
Average B-factors ($Å^2$)	22.74	21.75	34.08
R.m.s. deviations	0.61	0.16	0.21
Bond lengths (Å)	0.013	0.013	0.013
Bond angles (°)	1.64	1.63	1.64
Ramachandran statistics			
Most favoured/ Additional allowed	136 (96.45%)	146 (96.69%)	157 (96.21%)
Outliers	1 (0.71%)	1 (0.66%)	1 (0.61%)
PDB CODE	8A5N	8A2M	8A2L

†Rmerge= Σ hΣi |I(h,i)-<I(h)>|/ Σ hΣi I(h,i), where I(h,i) is the intensity of the ith measurement of reflection h and <I(h)> is the mean value of the intensity of reflection h. Criteria used in determination of resolution cut: Rpim <= 0.6000; I/σI >= 2.00; $CC_{(1/2)}$ >= 0.3000

* Values in parenthesis refer to highest resolution shell

Table S2. Structural features of the studied ferritins treated with SDS-based encapsulation protocol in comparison with untreated ferritin (5N27)*.

Protein	hHFt untreated downloaded from PDB	$hHFt_{SDS}$ obtained using the SDS-based encapsulation protocol	Ru1-$hHFt_{SDS}$ obtained using the SDS-based encapsulation protocol	TRIL- $hHFt_{SDS}$ obtained using the SDS-based encapsulation protocol
PDB CODE	5N27	8A5N	8A2M	8A2L
Total Accessible Surface Area (ASA) (Å^2)	9745.4	9863.5	9768.9	9622.8
ASA of backbone (Å^2)	882.3	873.4	858.8	879.1
ASA of side chains (Å^2)	8863.0	8990.1	8910.0	8743.7
Exposed nonpolar ASA (Å^2)	5347.4	5418.5	5466.0	5375.6
Exposed polar ASA (Å^2)	1939.3	1897.2	1853.8	1897.3
Exposed charged ASA (Å^2)	2458.7	2547.8	2449.1	2349.9
Fraction nonpolar ASA	0.55	0.55	0.56	0.56
Fraction polar ASA	0.20	0.19	0.19	0.20
Fraction charged ASA	0.25	0.26	0.25	0.24
% side ASA hydrophobic	18.87	18.84	19.02	19.47
Total volume (packing) (Å^3)	23715.7	23906.4	23624.4	23698.3

*determined using Vadar Server

Table S3

Mass signals recorded in the MALDI spectra of the tryptic digest of carboxyamidomethylated peptide obtained after encapsulation (A) and crystallization (B). The corresponding peptides are reported.

Theoretical m/z	Experimental m/z		Peptide sequence
	A	B	
245.18	245.19	245.09	IL(-)
480.26	480.24	480.24	WFK
536.32	536.32	536.32	FLTR
982.59	982.48	982.58	(-)FVKWFKK
